# Spatial clusters of suicide in the municipality of São Paulo 1996–2005: an ecological study

**DOI:** 10.1186/1471-244X-12-124

**Published:** 2012-08-23

**Authors:** Daniel H Bando, Rafael S Moreira, Julio CR Pereira, Ligia V Barrozo

**Affiliations:** 1 University Hospital, University of São Paulo Medical School, São Paulo, Brazil; 2 Department of Public Health, Aggeu Magalhães Institute, Oswaldo Cruz Foundation, Ministry of Health, Recife, Brazil; 3 Department of Epidemiology, School of Public Health, University of São Paulo, São Paulo, Brazil; 4 Department of Geography, School of Philosophy, Literature and Human Sciences, University of São Paulo, São Paulo, Brazil

## Abstract

**Background:**

In a classical study, Durkheim mapped suicide rates, wealth, and low family density and realized that they clustered in northern France. Assessing others variables, such as religious society, he constructed a framework for the analysis of the suicide, which still allows international comparisons using the same basic methodology. The present study aims to identify possible significantly clusters of suicide in the city of São Paulo, and then, verify their statistical associations with socio-economic and cultural characteristics.

**Methods:**

A spatial scan statistical test was performed to analyze the geographical pattern of suicide deaths of residents in the city of São Paulo by Administrative District, from 1996 to 2005. Relative risks and high and/or low clusters were calculated accounting for gender and age as co-variates, were analyzed using spatial scan statistics to identify geographical patterns. Logistic regression was used to estimate associations with socioeconomic variables, considering, the spatial cluster of high suicide rates as the response variable. Drawing from Durkheim’s original work, current World Health Organization (WHO) reports and recent reviews, the following independent variables were considered: marital status, income, education, religion, and migration.

**Results:**

The mean suicide rate was 4.1/100,000 inhabitant-years. Against this baseline, two clusters were identified: the first, of increased risk (RR = 1.66), comprising 18 districts in the central region; the second, of decreased risk (RR = 0.78), including 14 districts in the southern region. The downtown area toward the southwestern region of the city displayed the highest risk for suicide, and though the overall risk may be considered low, the rate climbs up to an intermediate level in this region. One logistic regression analysis contrasted the risk cluster (18 districts) against the other remaining 78 districts, testing the effects of socioeconomic-cultural variables. The following categories of proportion of persons within the clusters were identified as risk factors: singles (OR = 2.36), migrants (OR = 1.50), Catholics (OR = 1.37) and higher income (OR = 1.06). In a second logistic model, likewise conceived, the following categories of proportion of persons were identified as protective factors: married (OR = 0.49) and Evangelical (OR = 0.60).

**Conclusions:**

This risk/ protection profile is in accordance with the interpretation that, as a social phenomenon, suicide is related to social isolation. Thus, the classical framework put forward by Durkheim seems to still hold, even though its categorical expression requires re-interpretation.

## Background

Suicide is a major problem in public health: among people aged 15–44 years, it is the fourth leading cause of death worldwide [1]. According to the World Health Organization [2] the global suicide rate has been increasing since 1950. Estimates for 2020 based on current trends indicate that approximately 1.53 million people will commit suicide and 10–20 times more people will attempt suicide worldwide [3]. The emotional impact on families and friends affected by suicides or by attempted suicides can last many years.

Suicide trends are on the rise among the world’s emerging economies. In Brazil, annual suicide rates rose from 4.4 to 5.7 per 100,000 between 1980 and 2006 [4]. Nevertheless, there are significant differences among Brazilian regions, not only in figures but also in trends, as shown by Brzozowski [5] in a recent study covering the 26 Brazilian states. In the state of São Paulo, rates are lower and/or levelling off, which warrants monitoring because changes in social formation are likely to change suicide profile as well.

Since the end of the nineteenth century, it has been understood that suicide presents geographic and temporal variations [6,7]. In 1897, Durkheim constructed a framework for the analysis of the suicide, which still allows international comparisons amongst recent studies using the same basic methodology. He mapped suicide rates and realized that they clustered in geographic space. He also mapped alcoholism, the size of families and wealth, which overlapped the suicide rates map in northern France. In fact, a recent study using the same suicide data and geographical information system (GIS) techniques found a spatial cluster of high suicide rates in the north [8].

Durkheim's theory is based on two concepts: social integration and social regulation. Suicidal behaviour is common in societies where there is a low degree of social integration (egoistic suicide). The individual is protected from egoism by religions with strong group ties (e.g. Catholic Church) and family ties (e.g. married people). Suicidal behaviour is also common in societies where there is a low degree of social regulation (anomic suicide). Social regulation can be understood as external regulatory forces on the individual (economic cycles, income level) [7,9]. When Durkheim claimed that "poverty protects against suicide," he based on French departments and European countries observations. He noted higher suicide rates in wealthy regions. This finding raised the hypothesis that economic development could be related to individualism and, ergo, to social isolation and suicide. The other extremes of egoistic suicide and anomic suicide (altruistic suicide and fatalistic suicide, respectively) are also related to high rates of voluntary deaths, but were not generally applicable to modern western society [7,10]. As in modern society, integration rarely reaches excessive levels [9], weak social integration is more likely to affect the geographical patterning of suicide [8].

The geography of suicide is still poorly understood, with few studies that used maps to explore and represent the geographical variability and patterning of suicide [11]. In England and Wales, two main geographic patterns in young men (from 15–44 years old) were found [12]: a ‘bull’s-eye’ pattern, with highest suicide rates in the city centres of all ten of Britain’s largest cities and declined with increasing distance from the city centre and, a high rate of suicide in coastal areas. Otherwise, in non-Western nations as Taiwan, rates were highest in a mountainous rural area, with no evidence of above average rates in large cities [13].

Despite Durkheim’s findings, the relationship between socioeconomic characteristics and suicide is not straightforward. In France, Germany, Italy and England, for instance, different patterns have been observed since the early past century [14]. Today, it is known that the relationship between suicide and wealth is more complex. In developed and wealth countries (e.g., United States, Great Britain, Japan, France), suicide occurs more frequently in poor areas. Suicide trends in these countries are also either decreasing or stable [15]. These findings are evidence that the distribution of suicide has been changing along space and time.

The risk factors at individual level can be grouped in: a) distal factors - as genetic loading, personality characteristics, restricted fetal growth and perinatal circumstances, early traumatic life events, neurobiological disturbances and, b) proximal factors - as psychiatric and physical disorders, psychosocial crisis, availability of means and exposure to models [16]. However, at the contextual level there is no consensus yet to support the association between suicide and socio-economic characteristics of geographic areas [17]. Some studies report a direct relationship between suicide and income [18], while others observe this contrariwise [19] or yet fail to detect any association at all [20].

According to Susser [21] contextual characteristics are fundamental to understand how context affects the health of persons and groups since measures of individual attributes cannot detect the processes involved in relations as selection, distribution, interaction, adaptation, and other responses. This lay stress on ecological studies which, though not best fit to aetiological studies, do suit most properly studies with broader approaches. Suicide is an avoidable cause of death, which varies according to the socio-economic and cultural context of each society. Thus, understanding the relations among these phenomena is essential to define approaches that promote its occurrence reduction.

The Synthesis of Social Indicators of 2002 published by the Brazilian Institute of Geography and Statistics [22] states that one of the most remarkable characteristic of Brazilian society is social inequality. This can be observed in the city of São Paulo. According to the map of social exclusion/inclusion of the city, the districts of worse living conditions are in the outskirts, in contrast with well-off districts in the downtown area [23]. São Paulo is one of the most populous cities of the world; the last National Census estimated the population as 10.8 million inhabitants, with an elevated degree of urbanization [24]. The city of São Paulo is the major financial and economic center of Brazil. Although the São Paulo Metropolitan Region (38 surrounding municipalities) has the Gross Domestic Product (GDP) corresponding to 16.7% of total Brazilian GDP [25], 83.5% of the inhabitants have living conditions below a desirable standard, with low income, poor access to education, sanitation and housing [23].

From 1996 to 2005, 4,275 suicide deaths occurred in the city of São Paulo, with a mean suicide rate of 4.1/100,000 inhabitant-year. Revisiting the seminal ideia of Dukrheim's study, the present study aims to seek spatial patterns in the distribution of suicide rates, and then, verify its possible associations with socio-economic and cultural characteristics. Results should hopefully contribute to the better understanding of this phenomenon and thus advise prevention programs.

## Methods

An ecological study was conceived using the 96 Administrative Districts that comprise the whole municipality as the analysis unities. The first stage consisted of an exploratory spatial analysis of all suicide deaths of residents in the municipality of São Paulo, occurred from 1996 to 2005. We chose the data from 1996 onwards to avoid bias, since during this period the 10^th^ revision of the International Classification of Diseases and Deaths (ICD-10) was implemented. Mortality data were obtained from the Death Records Improvement Program (*Programa de Aprimoramento de Informação de Mortalidade* - PROAIM), the official health statistics source for the municipality. Suicide deaths corresponded to “intentional self harm”, codes X60 to X84, according to the ICD-10. The population estimates for each district were calculated by year by the São Paulo State System for Data Analysis Foundation (SEADE). Mortality and population data are publicly available and aggregated by Administrative District.

To elicit the putative spatial pattern of the suicide in the municipality, relative risks per administrative unit were calculated and mapped. As the suicide incidence is higher amongst males in the young and old age groups, relative risks were calculated accounting for the respective co-variates gender and age with the use of the software SaTScan [26]. A spatial scan statistical test was also performed through SaTScan to identify possible significantly high and/or low clusters of suicide. The hypothesis tested was of no departures from expected values in a bicaudal test using the Poisson distribution. The spatial scan statistics arranges a circular window of variable size in the map surface and allows its center to move in such a way that, for a given position and size, the window includes a different set of near neighbors. If the window includes a neighbor centroid, the whole district area is considered included. Statistical significance of a given cluster was ascertained by a likelihood ratio test using Monte Carlo procedures. The null-hypothesis was rejected when p <0.05 for the most likely cluster and p <0.02, for the secondary clusters.

The second step of the analysis consisted in verifying possible associations between the suicide risk clusters and socio-economic and cultural variables through logistic regression. The dependent logit variable contrasted risk cluster districts against the remaining non-risk cluster districts. The choice of independent variables to be scrutinised hinged on both Durkheim’s originals and the risk factors for suicide highlighted by WHO [1], PAHO/WHO et al. [27] and other reviews [17,28,29]. These data were obtained from a National Census of 2000 database as the following:

· Marital status, which comprised three categories: single, separated and married.

· Income: monthly average income per household (measured in number of minimum wages, Brazilian Real – R$).

· Migration and recent migration: people who lived in other municipality and people living in São Paulo less than five years.

· Religion: none, Catholic, Evangelical, Spiritualist.

Income is strongly correlated with education (Pearson Correlation was: +0.744; p-value < 0.01). So, we decided to use the average income, because it is one of the most common socioeconomic indicators used in this study design and allows comparisons with other studies. Each of these variables was at first analysed through simple logistic regression to ascertain significance as well as to discriminate categories related to risk from those related to protection. Provided that at least a 20% level of significance was achieved, these variables were considered for one of two multiple logistic regressions: one concerning risk factors and another concerning protective factors. A 5% significance level was established, and Hosmer and Lemeshow test was used to check model adjustment. Statistical analyses were performed through SPSS.

## Results

**Figure 1:**
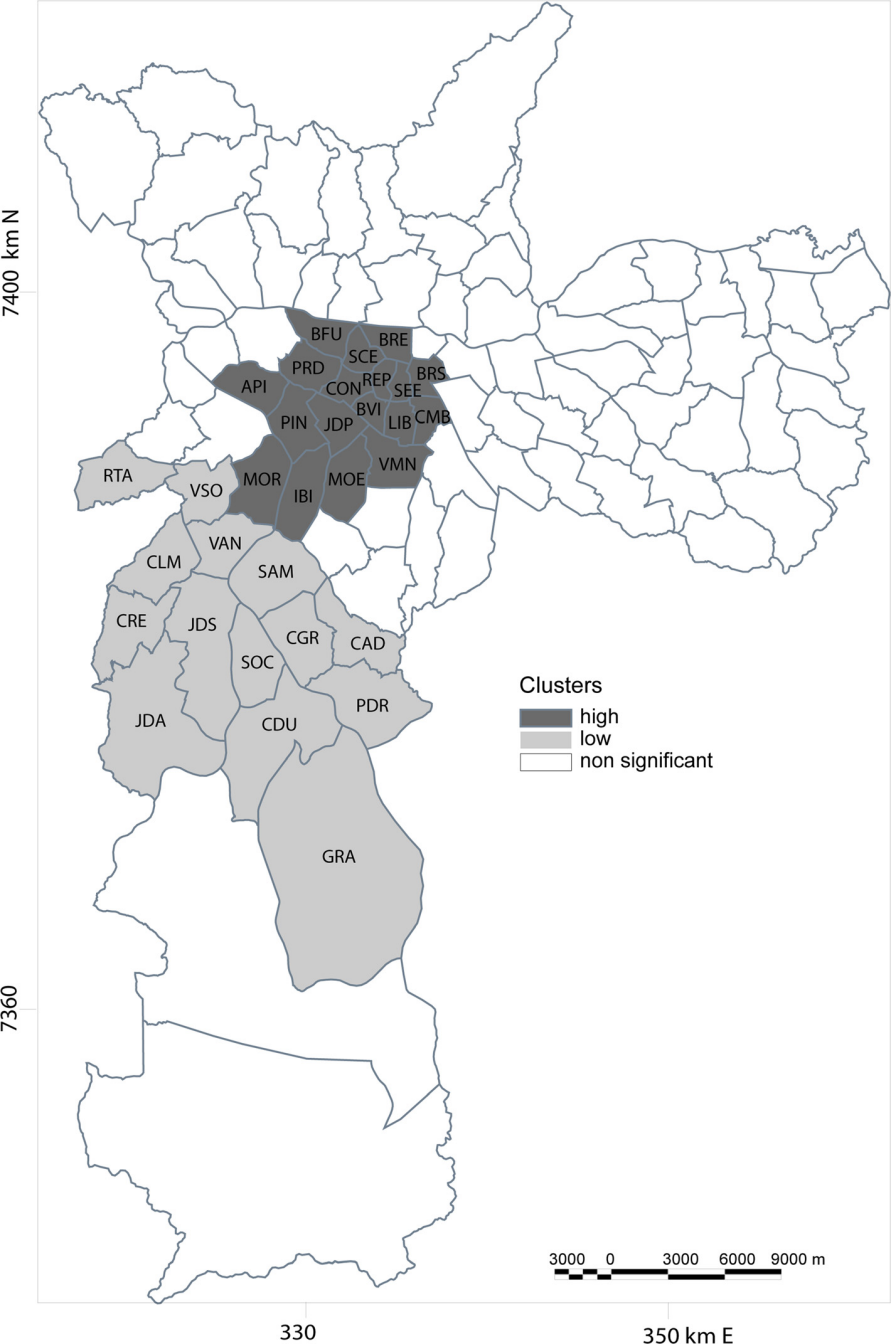
**Suicide spatial clusters of increased and decreased risk in the city of São Paulo, from 1996 to 2005.** Districts: API: Alto de Pinheiros, BFU: Barra Funda, BVI: Bela Vista, BRE: Bom Retiro, BRS: Bras, CDU: Cidade Dutra, CMB: Cambuci, CGR: Campo Grande, CLM: Campo Limpo, CRE: Capão Redondo, CAD: Cidade Ademar, CON: Consolação, GRA: Grajau, IBI: Itaim Bibi, JDA: Jardim Angela, JDP: Jardim Paulista, JDS: Jardim São Luis, LIB: Liberdade, MOE: Moema, MOR: Morumbi, PDR: Pedreira, PRD: Perdizes, PIN: Pinheiros, RTA: Raposo Tavares, REP: Republica, SCE: Santa Cecilia, SAM: Santo Amaro, SEE: Se, SOC: Socorro, VAN: Vila Andrade, VMN: Vila Mariana, VSO: Vila Sonia.

Two significant spatial clusters were identified. One cluster classified as high risk presented 6.3 suicides per 100,000 inhabitants with relative risk of 1.66. This cluster is located downtown, the south-central and western-central area of the city and includes 18 Administrative Districts. A secondary cluster classified as low risk was also identified with 3.3 suicides per 100,000 inhabitants and a relative risk of 0.78 (Figure 1).

The multiple regression analysis assessed effects of both risk and protective factors as shown in the Tables 1 and 2. Both models achieved a high level adjustment in the Hosmer and Lemeshow test with p = 0.991 and p = 1.000 respectively.

**Table 1:** Multiple logistic regression measuring effects of risk factors to High Risk Suicide cluster

**Variable**	**β**	**p**	**OR**	**95% CI**
Marital status				
Single	0.859	0.031	2.36	1.081 – 5.150
All others			1	
Migrant				
Yes	0.403	0.002	1.497	1.156 – 1.937
No			1	
Religion				
Catholic	0.312	0.034	1.366	1.024 – 1.823
All others			1	
Income (n° of minimal wages)	0.054	0.026	1.056	1.007 – 1.107

**Table 2:** Multiple logistic regression measuring effects of protective factors to High Risk Suicide cluster

**Variable**	**β**	** *p* **	**OR**	**CI 95%**
Marital status				
Married	−0.72	0.003	0.487	0.302 – 0.786
All others			1	
Religion				
Evangelical	−0.507	0.013	0.603	0.404 – 0.899
All others			1	

As shown in Table 2, one must consider the nature of the variables to properly compare their effects. Income, though it displayed the least OR values, does have a considerable impact on the likelihood of its association with high suicide risk clusters. Income values ranged from 1.2 to 100.3 times the minimum wage with a mean of 24.4 and a standard deviation of 19.95. Thus, an income increase of as little as five, or approximately ¼ of the variation pattern, raised the OR figures from 1.056 to 1.28. In other words, a very modest increase of one minimal wage level in income increased the chances of suicide in a high-risk cluster by 5.6%, and an increase of five minimal wages levels increased the chances by 31%.

## Discussion

The discovery of a significant suicide risk cluster represents an important point for further investigating the risk factors for suicide. In the case of the city of São Paulo, statistical analysis revealed that the relative risk of suicide is higher in the downtown, south-central and western-central areas of the city. Although the average rate for the entire city may be considered low (< 5.0 per 100,000 inhabitants) according to Diekstra and Gulbinat [30], locally, the very downtown area of the city presented a rate of 6.3 per 100,000 inhabitants, a medium rate in this classification. This discovery warrants enhanced attention for public health action in these areas. It is important to mention that in the cluster detection procedure with a circular window in large areas with spatial variations in the population density, the resulting clusters can include districts with lower risk. Despite this limitation, in a recent review of software for space-time disease surveillance, SaTScan was highlighted as the most developed and robust software for cluster detection [31].

Multiple logistic regressions pointed out that marital status plays an important role in suicide; even though estimates present wide confidence intervals, the results suggest that being single more than double the likelihood (OR = 2.36) in high suicide risk clusters whereas being married almost halves such risk (OR = 0.49). Durkheim was one of the first to observe that high suicide rates were associated with being single in France, and his theory could be used to explain this association in São Paulo. According to Durkheim’s theory, suicide varies inversely with the degree of integration of the individual to his social group. The spatial distribution of the proportion of singles (41.06%) and separated (18.61%) in the central region of the city of São Paulo is greater than the whole city (38.91% and 12.91%, respectively), which could lead one to interpret these suicides as similar to ‘selfish suicides’ in Durkheim’s work. Recent study based on suicidal individuals from São Paulo, from 1996–2008, confirmed that being single is significant as risk factor for suicide [32].

In a review of 84 papers from 1981 to 1995, Stack [28] concluded that more than three quarters of the studies supported the protective factor of being married. This was in agreement with his previous analysis of 15 nations (mostly European) in which he found that marriage lowered suicides for both males and females [33]. A study of 12 developed countries with data from the end of the twentieth century, including the United States, Australia and European countries, identified marriage as suicide protective factor [34]. The same effect was observed in Italy with data from 2000 to 2002 [35].

Data from Unites States between 1986 and 2002 showed that larger families were associated with lower suicide rates whereas divorced or separated, widowed, or never married individuals conditions were associated with higher suicide rates though only among men [36]. In Northern Ireland, from 1996 to 2005, marriage was found to protect both genders against suicide [37]. Another study verified protection only for married men in Austria using data from 1970 to 2001 [38]. A study with recent Taiwan data observed that never married males had the highest suicide rates [39]. Divorce was also related to suicides in Japan [40], Taiwan [41], Australia [42] and Romania [43].

The migrant condition was found to increase the likelihood in high suicide risk clusters in almost 50% (OR = 1.50) of São Paulo. The WHO’s “Self-directed violence” report [1] alerts that suicide rates in a given migrant group have been found to be similar to that of the migrant’s country-of-birth. Indeed, Voracek et al. [44] retrieved a meta-analysis of 33 studies with data from seven host countries, and a strong and direct association was found between immigrant rates and country-of-birth rates. The city of São Paulo still attracts a great number of migrants from all Brazilian regions [25] whereas migration from other countries is also rising. In the downtown area, in the high suicide risk region of the city, migrants correspond to 79.8% of residents in contrast with only 13.2% of migrants in the city as a whole. Unfortunately, no information regarding the migrants’ origins is available to provide a control for this association. Whether this is due to imported patterns or to locally developed behaviours, the migrant condition may be a flag for suicide risk, which should at least invite health surveillance.

The Catholic condition was found to increase the likelihood in high suicide risk clusters in 37% (OR = 1.37). Though at first this seems to conflict with Durkheim’s original finding that Protestants were more prone to suicide, one must understand that he attributed this finding to Protestants’ being less socially integrated. In São Paulo, the Catholic group is known to be less socially cohesive than Evangelical groups [45] and thus may be susceptible to a higher suicide risk. In recent decades, the national census has shown a decrease in the number of Catholics in São Paulo (78.9 to 67.1%) and an increase of Evangelicals (8.54 to 16.4%) [45,46]. Evangelicals have greater popular appeal. The number of Evangelical churches is three times the Catholic, in poor areas of the outskirts. Among the Evangelicals, there are several social networks that reduce their vulnerabilities [45], thereby decreasing the suicide rates. Nevertheless, Prandi et al. [47] showed a relationship between Evangelicals and poverty and found that the geographic distribution of these two variables was concentrated in the periphery of the city. In the state of Rio de Janeiro, Brazil, a recent ecological study found that being Evangelical is a protective factor for suicide [48].

Neither income itself nor its putative interaction with religion achieved statistical significance in multiple logistic regression (Table 2); therefore, only the crude protective effect of being Evangelical against suicide risk was detected. Regarding the results in Tables 1 and 2, it may be possible to reconcile the observations found in the present study and in the one by Prandi et al. [45,47]. The lower the income or the higher the proportion of Evangelicals, the less the risk (Table 2).

In the present study, the chances of a high suicide risk cluster increased by 5.6% with each increase of one minimal wage level in income. In northern India, from 1996–2005, income was inversely related to suicide [49]. Another study using World Health Organization (WHO) data focused on countries with a medium Human Development Index (HDI) and found that education and telephone density were directly related to suicide while a high Gini index was inversely related to suicide [29]. Other recent ecological studies performed in the United States [50], Japan [51], Australia [52], Northern England [53], Finland [54,55] and Tuscany (Italy) [56] identified an inverse relationship between income and suicide. Another study carried out in Italy suggested that regions with higher economic status have the highest suicide rates [57]. Nevertheless, the relationship between suicide and wealth is far more complex because it relates to multiple socio-economic factors resulting from numerous historical and cultural elements inherent in each population. Accordingly, there are multiple divergent findings in the literature regarding the direction of this association (e.g., direct, inverse or no relation) between wealth and suicide. The manual of suicide prevention by PAHO/WHO et al. [27] considers the extremes of economic strata as suicide risk factors. Many factors can contribute to this wide variation of findings including the size of the aggregated population, metrics of socio-economic characteristics and the inclusion of different potentially confounding variables, study designs, secular trends, and cultural aspects [17].

The present study has some limitations. The first is inherent to the ecological study design. The association observed between variables at the group level does not necessarily represent the association that exists at the individual level. This bias is known as *ecological fallacy*. Other limitation is related to data collection due to misclassification of suicide. Different procedures and cultural and social practices and values probably have various effects on death records and lead to misclassification of suicide [1,16]. Among those is the fact that health insurance does not cover health expenses when injury or death is resulted of suicide attempt, which can lead to underestimation.

The results herein presented are in agreement with Durkheim’s original suggestion that poverty had a protective effect against suicide [17]. This could suggest that economic development leads to individualism that, in turn, leads to higher suicide rates [15]. Thus, to better understand the role of wealth, one should seek a better understanding of poverty as suggested by Paugam [58], whose work was based on Castel’s [59] work distinguishing between disqualifying poverty and integrated poverty. The latter, which best suits São Paulo, is found in societies where low life standards are compensated by a solidarity response within family, neighbourhood, and region.

## Conclusions

The present study used an ecological approach similar to that utilized by Durkheim. Criticisms applicable to his study are likewise applicable to the present study. None of the covariates of suicide herein presented can or should be interpreted as causes in the natural history of the phenomenon of suicide. Indeed, suicide may not be a condition of which one should seek etiopathological paths. Medicine is concerned with individuals, whereas public health is focused on collections of individuals. The present study has identified clusters of high suicide risk as well as their correlates, and this should assist in the planning, intervention, monitoring and evaluation of the phenomenon of suicide.

## Competing interests

The authors declared that they have no competing interests.

## Authors’ contributions

Conceived and designed the study: DHB and LVB. DHB and LVB undertook the spatial scan statistic and DHB and RSM, the statistical analysis. DHB drafted an initial manuscript. DHB, LVB and JCRP contributed to writing the final manuscript. All authors read and approved the final manuscript.

## Pre-publication history

The pre-publication history for this paper can be accessed here:

http://www.biomedcentral.com/1471-244X/12/124/prepub
